# SNP Array in Hematopoietic Neoplasms: A Review

**DOI:** 10.3390/microarrays5010001

**Published:** 2015-12-22

**Authors:** Jinming Song, Haipeng Shao

**Affiliations:** Department of Hematopathology and Laboratory Medicine, H. Lee Moffitt Cancer Center and Research Institute, 12902 Magnolia Drive, Tampa, FL 33612, USA; Jinming.Song@moffitt.org

**Keywords:** SNP array, hematopoietic, myelodysplastic syndrome, leukemia, lymphoma

## Abstract

Cytogenetic analysis is essential for the diagnosis and prognosis of hematopoietic neoplasms in current clinical practice. Many hematopoietic malignancies are characterized by structural chromosomal abnormalities such as specific translocations, inversions, deletions and/or numerical abnormalities that can be identified by karyotype analysis or fluorescence *in situ* hybridization (FISH) studies. Single nucleotide polymorphism (SNP) arrays offer high-resolution identification of copy number variants (CNVs) and acquired copy-neutral loss of heterozygosity (LOH)/uniparental disomy (UPD) that are usually not identifiable by conventional cytogenetic analysis and FISH studies. As a result, SNP arrays have been increasingly applied to hematopoietic neoplasms to search for clinically-significant genetic abnormalities. A large numbers of CNVs and UPDs have been identified in a variety of hematopoietic neoplasms. CNVs detected by SNP array in some hematopoietic neoplasms are of prognostic significance. A few specific genes in the affected regions have been implicated in the pathogenesis and may be the targets for specific therapeutic agents in the future. In this review, we summarize the current findings of application of SNP arrays in a variety of hematopoietic malignancies with an emphasis on the clinically significant genetic variants.

## 1. Introduction

**Figure 1:**
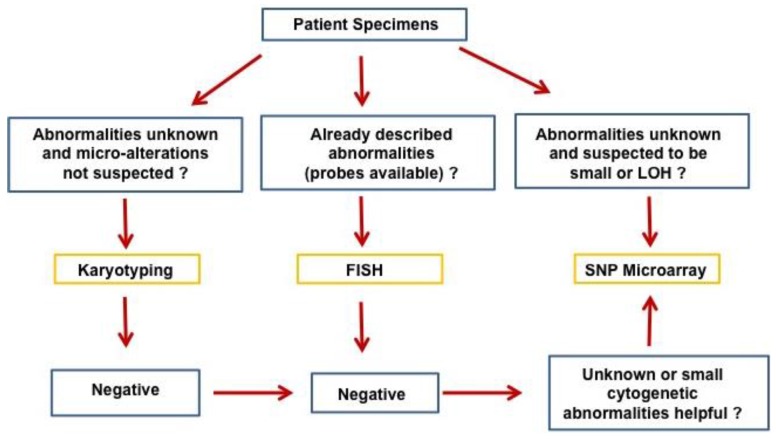
Proposed application of SNP array in hematopoietic malignancies.

The progressive accumulation of genetic changes plays an essential role in the tumorigenesis and evolution of human cancers. The genetic changes commonly seen in human cancers include chromosomal translocations, amplifications, allelic loss, loss of heterozygosity, deletions, mutations, and epigenetic changes/DNA methylation affecting oncogenes and tumor suppressor genes [1]. The resolution of genetic alterations identified in clinical specimens has been pushed to the single nucleotide level over the decades with advancements in genetic technologies. Conventional cytogenetic analysis with G-banding karyotyping, a routine clinical analysis in cytogenetic labs, allows differentiation of approximately 400–500 bands per haploid genome [2]. At this level of resolution, chromosomal change over 10 Mb can be detected. Fluorescence *in situ* hybridization (FISH) offers high sensitivity and specificity of detecting genetic abnormalities such as translocations, aneuploidy, deletions, inversions, or amplifications by using DNA probes targeted to known DNA sequences [3]. FISH can identify genetic changes at a resolution up to a few kilobases (kb), but is not suited for identification of unknown genetic changes or global chromosomal abnormalities. Array-based comparative genomic hybridization (aCGH) developed in the early 1990s offers efficient high-throughput analysis of the entire genome for identification of copy number variations/aberrations (CNVs/CNAs) that are usually not detectable by conventional karyotyping or targeted FISH studies, and has an improved resolution down to 100 kb [4,5,6,7]. Single nucleotide polymorphism (SNP) arrays, manufactured by Affymetrix and Illumina, were initially designed for high-throughput SNP genotyping, but were quickly applied to cancer genomics [8,9,10,11]. In contrast to aCGH, SNP arrays are able to detect both CNVs/CNAs and loss of heterozygosity (LOH) or copy-neutral LOH/uniparental disomy (UPD), which are frequently involved in the development of cancers. With the advance in technology and marked improvements in resolution, the new SNP array offers over 90% coverage of known copy number variants by using more than 946,000 probes and an average inter-marker distance of 680 base pairs. This high level of resolution of cytogenetic changes has only recently been surpassed by next generation sequencing (NGS) technology developed in the last decade [12,13]. Ever since the invention of SNP arrays, they have been extensively applied to various hematologic malignancies. While currently there are no clinical guidelines on the use of SNP array in hematopoietic malignancies, SNP array will certainly be useful in difficult cases, especially in myelodysplastic syndrome (MDS) diagnosis, when other methodologies fail to identify cytogenetic abnormalities. A proposed flow chart for the application of SNP array in hematopoietic malignancies is presented in Figure 1. In this review, we summarize the important findings of chromosomal changes in hematopoietic malignancies identified by SNP array analysis.

## 2. Acute Lymphoblastic Leukemia/Lymphoma

Acute lymphoblastic leukemia/lymphoma is the most frequent pediatric malignancy, affecting 20–40 patients per million children in developed countries [14], and accounts for 20% of all acute leukemias in adults. B-lymphoblastic leukemia/lymphoma (B-ALL) is the most common type of acute lymphoblastic leukemia, and comprises genetically distinct subtypes including B-ALL with Philadelphia chromosome t(9;22)(q34;q11.2) (*BCR-ABL1*), t(v;11q23) (*MLL* rearranged), t(12;21)(p13;q22) (*TEL-AML1*), t(5;14)(q31;q32) (*IL3-IGH*), t(1;19)(q23;p13.3) (*E2A-PBX1*), hyperdiploidy, hypodiploidy, and about 25% cases without defined cytogenetic abnormalities [15]. Pediatric B-ALL has a favorable prognosis with approximately 80% rate of cure, while adult B-ALL has an inferior prognosis with about 40% rate of cure [16,17,18,19,20,21,22,23,24]. B-ALL with t(9;22)(q34;q11.2) (*BCR-ABL1*) is associated with the worst prognosis in both children and adults. Studies have shown that the currently identified chromosomal translocations are early initiating genetic events but are not sufficient to induce ALL [25].

To identify additional genetic lesions important for leukaemogenesis, a SNP array is well-suited for global genomic mapping of B-ALL. Irving *et al.* [26] first applied The Affymetrix 10K SNP array with resolution of 100 to 200 kb was used in 10 cases of pediatric B-ALL and demonstrated the usefulness of this technique in studying B-ALL. Of the 10 cases, LOH was detected in eight cases with the most frequent abnormality (50%) in chromosome 9p harboring the *CDKN2A/B* (*INK4*) gene locus. The loss of *INK4* gene locus was only observed at relapse in three of the four cases, suggesting its association with treatment failure. Subsequently, Mullighan *et al.* [27] performed the first large-scale study of 242 cases of paediatric ALL, including 192 B-ALL and 50 T-ALL, by using Affymetrix SNP arrays that examine over 350,000 loci with an average resolution of less than 5 kb. Matched remission samples allowed the identification of somatic CNAs and LOH in leukemic blasts. The SNP arrays showed a low number of somatic copy number alterations (mean of 6.46) per case in ALL, with deletions outnumbering amplifications almost 2:1. The frequency of CNAs varied significantly between different cytogenetically defined ALL subtypes, with deletions more frequent than gains of DNA. Chromosomal deletions occurred more frequently in B-ALL with *ETV6–RUNX1* and hypodiploidy with average of six deletions per case, up to 21 deletions, and only one deletion in *MLL* rearranged B-ALL. Gains of DNA occurred most frequently in hyperdiploid B-ALL (average of 10 gains), and uncommon in other types of ALL. The study identified 54 recurring regions of deletion that were mostly focal with the minimal deletion less than 1 Mb, and 24 deletions harboring only one single gene. The most important finding was that genes regulating normal B-cell development were deleted or mutated in approximately 40% cases of B-ALL. Copy number changes of *PAX5*, which is essential for B-cell differentiation, occurred in about 30% cases of B-ALL, making *PAX5* the most frequently altered gene in B-ALL. These changes resulted in either reduced level or hypomorphic alleles of *PAX5*. Sequencing studies also identified a variety of somatic mutations in *PAX5* resulting in either lost or altered DNA-binding or transcriptional functions. Other important genes deleted in ALL included *EBF1*, *TCF3*, *LEF1*, *IKZF1* (*IKAROS*), and *IKZF3* (*AIOLOS*). These findings suggest that ALL is not a neoplasm characterized by chromosomal instability, and genetic alterations in genes controlling B-cell development (*PAX5*, *EBF1* and *IKZF1*) are common and play important roles in B-ALL leukaemogenesis. In a separate study, Kawamata *et al.* [28] studied 399 pediatric ALL samples with matched remission marrow using Affymetrix 50K SNP arrays, and identified three most common genetic alterations: deletion of *ETV6*, deletion of *CDKN2A/p16INK4A*, and hyperdiploidy. This study also confirmed the deletions of *PAX5* (9p13), *EBF* (5q33), *IKAROS* (7p12.2), *AIOLOS* (17q12), *LEF1* (4q25), *RAG1* (11p12), and *RAG2* (11p12) in pediatric ALL, albeit with a lower frequency except *PAX5*. Uniparental disomy (UPD) was frequently identified, especially in chromosome 9. In addition, hyperdiploid ALL without gains of chromosomes 17 and 18 was found to have poor prognosis. The common deletions of *CDKN2A* at 9p21 (29%) and *ETV6* (*TEL*) at 12p13 (3/24, 12%) were also confirmed by Bungaro *et al.* [29] in a separate study.

T-ALL comprises about 25% cases of adult ALL and approximately 15% cases of childhood ALL and is most commonly present in adolescents as mediastinal lymphoma. The most frequent recurrent cytogenetic abnormalities involve translocation of T-cell receptor gene locus (14q11.2, 7q35, and 7p14-15) with a variety of partner genes such as *HOX11*, *MYC*, *TAL1*, *RBTN1/2*, and *LYL1* [30,31] and, thus, T-ALL is genetically more heterogeneous. In their SNP array analysis of 50 cases of T-ALL, Mullighan *et al.* [32] identified multiple new genomic changes in T-ALL, including deletions of *TAL1*, *RB1*, and *PTEN*, and duplications of protooncogene *MYB*. Most recently, Karrman *et al.* [33] investigated 47 cases of T-ALL with an Illumina HumanOmni1-Quad BeadChip containing >1 million markers and a median marker interval of 1.5 kb. Copy number changes and UPD were identified in the majority of cases (92%), with a median of three changes/per case. This study identified recurring region of deletion harboring genes *CDKN2A*, *CDKN2B*, *LEF1*, *PTEN*, *RBI*, and *STIL*. In terms of uniparental disomy (UPD), the T-ALL lacked whole chromosome UPD, but showed segmental UPDs (sUPDs) in 42% of cases, with a high proportion of sUPD 9p (30% of the cases) associated with homozygous *CDKN2A* deletion. Therefore, disruption of the *p53* and *RB1* pathways through deletion of *INK4/ARF* on *CDKN2A* gene locus appears to be important in the pathogenesis of T-ALL.

The genomic changes that may explain the difference in survival between pediatric and adult ALL were addressed by Okamoto *et al.* [34] with Affymetrix 50K or 250K SNP arrays. The authors studied 75 cases of adult ALL and 399 cases of pediatric ALL. This study showed 572 genomic alterations with a mean of 7.6 genomic changes per case in adult ALL. The genomic changes in adult ALL were comparable to those identified in pediatric ALL, including deletions of 3p14.2 (*FHIT*), 5q33.3 (*EBF*), 6q, 9p21.3 (*CDKN2A/B*), 9p13.2 (*PAX5*), 13q14.2 (*RB1*), and 17q11.2 (*NF1*). The recurrent genomic alterations had similar rate of occurrence in pediatric and adult ALL. As the adult ALL cases were all non-hyperdiploid, the pediatric ALL cases were divided into hyperdiploid (HD) and non-hyperdiploid groups. There was no significant difference between adult and non-HD pediatric ALL in terms of deletions of 3p14.2 (*FHIT*), 9p21.3 (*CDKN2A/B*), 9p13.2 (*PAX5*), 13q14.2 (*RB1*), and 17q11.2 (*NF1*), and adult ALL showed more frequent deletion of 17p (*TP53*) and duplication of 17q than non-HD pediatric ALL (11% *vs*. 2%, and 9% *vs*. 1%, respectively). Overall, there were no unequivocal CNAs identified by SNP array that can account for the differences of prognosis between adult and pediatric ALL. The characteristic deletions of *CDKN2A*, *PAX5*, *IKZF1*, *ETV6*, *RB1*, and *EBF1* genes were also identified by Safavi *et al.* [35] in adult ALL using SNP array covering 5 million markers and a resolution of 10 kb. A number of novel recurrent cryptic genetic changes involving *BCAT1*, *SERP2*, *RAB30*, *SRPR*, *ST3GAL4*, *ASS1*, *RASSF3*, *FUBP3*, *BCL11A*, *GAB1*, *LINGO2*, *TOX*, and *CXCR4* genes, and partial and whole-chromosome UPDs were also discovered in adult ALL. Their significance in pediatric and adult ALL remains to be determined.

SNP arrays were designed for genome-wide association (GWA) study, and it was naturally applied in ALL for germline SNPs that may have an association with ALL. Trevino *et al.* [36] studied 317 cases of pediatric ALL along with 17,958 control cases with an Affymetrix 500K Mapping array, and found two SNPs at chromosome 10q21 (rs10821936 and rs10994982) located in intron 3 of the *ARID5B* gene to be associated pediatric ALL. In addition, both SNPs discriminated hyperdiploid B-ALL from other major ALL subtypes. A genome-wide association study by Papaemmanuil *et al.* [37] on two case-control series with 907 ALL cases and 2398 controls also identified an association between a SNP at 10q21.2 in the *ARID5B* gene (rs7089424) and pediatric ALL. The 10q21.2 (*ARID5B*) risk association was selective for hyperdiploid B-ALL. In addition, they also found two additional risk loci for ALL at 7p12.2 (*IKZF1*, rs4132601), and 14q11.2 (*CEBPE*, rs2239633). The association between genetic variations at 7p12.2 (*IKZF1*), 10q21.2 (*ARIDB5*), and 14q11.2 (*CEBPE*) with pediatric ALL was replicated by Prasad *et al.* [38] in genotyping 1384 cases of pediatric B-ALL and 1877 controls. These findings indicate that common germline variants contribute to the risk of development of pediatric ALL. As both *ARID5B* and *IKZF1* play important roles in B-lymphocyte growth and differentiation, the possibility of SNPs in these genes predispose the patients to the development of B-ALL is high.

## 3. Acute Myeloid Leukemia

Acute myeloid leukemia (AML) is genetically heterogeneous, ranging from cases with recurrent cytogenetic abnormalities to approximately 40% cases with normal karyotype, some of which have prognostically significant somatic mutations. AMLs with t(15;17), inv(16) and t(8;21) respond well to chemotherapy and have good prognosis, while AMLs with Philadelphia chromosome t(9;22), complex karyotype, −5/−5q, −7/−7q, and 11q23 translocations have poor prognosis. In AMLs with normal karyotype, *FLT3* internal tandem duplications (ITDs) are associated with poor prognosis [39], while mutations of *NPM1* and *CEBPA* are associated with favorable prognosis [40]. In 2005, Raghavan *et al.* [41] first applied a 10K SNP array in 64 cases of AML and identified partial uniparental disomy (pUPD) in approximately 20% cases. A similar study with 10K SNP array demonstrated homozygous mutations involving genes *FLT3*, *CEBPA*, and *RUNX1* in approximately 50% patients with UPD involving corresponding chromosomal regions [42]. Subsequently, Gupta *et al.* [43] expanded the study with 10K SNP array on 454 cases of AML from young adults, and found nonrandom acquired UPD (aUPD) in 17% cases, preferentially affecting chromosomes 13q, 11p, and 11q, similar to the findings by Raghavan *et al.* [43], and additional recurrent aUPDs at 2p, 17p, 2q, 17q, 1p, and Xq. AMLs with *FLT3*-ITD had aUPD 13q involving the *FLT3* gene, while AMLs with *FLT3*-ITD-/*FLT3*-TKD+ mutation did not have aUPD 13q.

These early studies suffered from the low resolution of the SNP arrays available at that time. With advances in SNP array technology, Bullinger *et al.* [44] were able to use 50 and 500K Affymetrix SNP arrays on 157 cases of cytogenetically normal AML. The cohort showed 12% aUPDs with chromosomal regions 6p, 11p, and 13q most commonly affected and all aUPDs were >29 Mb. The aUPDs were associated with mutations in *NPM1* or *CEBPA*, which suggested that aUPDs may affect genes critical for hematopoiesis. In terms of aCNAs, as expected, the 500K SNP array was much more sensitive than 50K SNP array, which missed approximately 60%–70% of CNAs detected by the 500K SNP array. aCNAs were identified in 49% cases, with genomic losses (1.21/case) more frequent than gains (0.25/case). The recurrent genomic deletions included bands 3p14.1-p13, 6q27, 8q23.3, 10q11.21, 11q25, 12p13.2, and 15q21.3, that harbor genes *FOXP1* and *RYBP* (3p14.1-p13), *RPS6KA2* (6q27), *TRPS1* (8q23.3), *HNRPF* (10q11.21), *ETV6* (12p13.2) and *RFXDC2* (15q21.3). In a separate study, Walter *et al.* [45] applied a 1.85 million SNP array from Affymetrix in 86 adult patients with *de novo* AML, and identified a total of 201 aCNAs in 44% of the cases with a mean of 2.34 CNAs per genome. Acute erythroid leukemia and acute megakaryocytic leukemia had more CNAs (10–29 CNAs per genome) than other morphologic variants of AML. CNAs were detected in 24% AMLs with normal cytogenetics and in 40% AMLs with abnormal karyotype. 12 chromosomal regions (eight deletions and four amplifications) containing at least one gene implicated in AML or MDS (deletions of 3p14.1: *FHIT*, 5q31.1: *CTNNA1*, 12p12.3: *ETV6*, 16q22.1: *CBFB*, 17p13.1: *TP53*, 17q11.2: *NF1*, and amplifications of 8q23.2: *MYC*, 11q23.3: *MLL*, and 21q22.2: *ETS2*) were identified in multiple AML cases. CNAs in chromosomal regions 17q11.2 and 21q22.2 (CNAs spanning *NF1* and *ETS2*) were found in at least five cases, most of which had complex karyotypes, and associated with worse overall survival. aUPD was infrequent occurring in eight of 86 genomes, with most in AML with normal cytogenetics. 50% of the AML studied had no CNA or UPD at this resolution. The identified CNAs did not predict overall or event-free survival independent of cytogenetics. Radtke *et al* [46] showed similarly very low burden of genomic alterations in pediatric *de novo* AML, with a mean of only 2.38 somatic copy-number alterations per case. These studies indicate that AMLs are not genomically unstable and the genes implicated in these CNAs likely play important role in the leukemogenesis of AML.

While Walter’s study did not identify any prognostically significant CNAs or UPDs, three groups clearly showed prognostic significance of SNP array lesions in AML, likely due to utilization of more stringent SNP lesion detection algorithms. Parkin *et al.* [47] examined 114 previously untreated prospectively enrolled AML patients with Affymetrix SNP 6.0 arrays, and showed that ≥2 genomic lesions detected by SNP 6.0 array almost doubled the risk of death after controlling for age- and karyotype-based risk by multivariate analyses. *P53* mutations, or *P53* mutations coupled with 17p-LOH conferred an independent negative prognosis. Tiu *et al.* [48] performed 250 K and 6.0 SNP arrays on 140 cases of primary (p) and secondary (s) AML, and demonstrated that patients with genomic lesions including acquired somatic UPD identified by SNP array had worse overall survival (OS) and event-free survival (EFS) in pAML with normal cytogenetics and in pAML/sAML with abnormal cytogenetics. The SNP array lesions, AML type, and metaphase cytogenetics had independent predictive value for OS by multivariate analyses. In a study of 133 cases of AML with normal cytogenetics by Genome-Wide Human SNP 6.0 Array, Yi *et al.* [49] found at least one abnormal SNP lesion in 32.3% cases. Detection of abnormal SNP lesions by SNP-A karyotyping conferred an unfavorable prognosis for overall survival by multivariate analyses. All these studies confirmed the clinical relevance as well as prognostic significance of SNP lesions in AML, especially AML with normal cytogenetics, which would allow a better prognostic stratification of patients with AML for appropriate treatment.

## 4. Myelodysplastic Syndrome

Myelodysplastic syndrome (MDS) is a group of clonal hematopoietic neoplasm characterized by ineffective hematopoiesis, cytopenia, morphologic dysplasia, and potential progression to acute myeloid leukemia [50]. Most patients with MDS die of bone marrow failure rather than transformation to acute myeloid leukemia. MDS is diagnosed based on the World Health Organization (WHO) classification, but the prognosis for survival is stratified based on the international prognostic scoring system (IPSS) and revised IPSS (IPSS-R) that incorporate cytogenetic abnormalities, percentage of bone marrow myeloblasts, and number of cytopenias [51,52]. As part of the IPSS-R system, cytogenetic abnormalities are stratified from very good with single del(11q) and −Y to very poor with complex karyotype (>3 abnormalities). Approximately half of patients with MDS have normal karyotype that is associated with good prognosis in the IPSS-R system, but MDS patients with normal karyotype are still heterogeneous genetically. SNP arrays were therefore performed on MDS to identify additional occult genetic abnormalities, especially in patients with normal karyotype.

Gondek *et al.* [53,54] was the first group to study MDS with SNP array. They applied an Affymetrix 50K SNP Assay to 66 and 72 patients with MDS, and found chromosomal defects in 82% of MDS patients, including 68% patients with normal karyotype, 81% patients with abnormal karyotypes, with chromosomes 8, 7, 5, and 11 most frequently involved. Segmental uniparental disomy (sUPD) was found in 33% of patients with MDS, and usually in regions frequently affected by deletions detected by metaphase cytogenetic analysis, including 7q and 11q. In a similar study, Mohamedali *et al.* [55] studied 119 patients with low-risk MDS with 50 K, 250 K and 500 K SNP arrays and identified deletions in 10%, amplifications in 8%, and UPD in 46% of cases. Nowak *et al.* [56] showed that CNAs and LOH can be identified in the CD34 positive blasts. These early studies suffered from absence of paired normal tissue for each case, which made distinction of inherited CNAs and LOH from somatic acquired ones difficult. Heinrichs *et al.* [57] performed a prospective study of matched pairs of bone marrow and buccal cell (normal) DNA from 51 patients with MDS by 250K SNP array, and identified somatically acquired genomic abnormalities in 41% patients, including 15% in MDS with normal karyotypes. UPDs affecting chromosome 7q was associated with rapidly progressive clinical course despite a low-risk IPSS score [57]. Similarly, Tiu *et al.* [58] analyzed 250 cases of MDS by 250K and Affymetrix SNP Array 6.0 with paired bone marrow and CD3+ lymphocytes to distinguish germline lesions. In this study, they showed that combined metaphase cytogenetics and SNP array had a higher diagnostic yield of chromosomal defects (74% *vs.* 44%), compared with conventional karyotyping. The genetic abnormalities detected by SNP arrays were deletions and aUPD involving chromosomes 1, 5, 7, 11, 17, and 21. While Mohamedali’s study failed to show independent prognostic significance of the genetic lesions identified by SNP array on multivariate analysis, Tiu showed that the presence of new genetic lesions detected by SNP array was predictive of poor prognosis in MDS by univariate and multivariate analyses [58]. These studies proved the utility of SNP array in detecting submicroscopic genetic lesions in MDS as a complement to metaphase cytogenetics, and the lesions identified by SNP arrays can further help prognostic stratification of MDS patients.

MDS can be difficult to diagnose clinically due to many mimickers of the disease and the lack of significant morphologic dysplasia in a small subset of cases. For example, severe aplastic anemia (AA) may be difficult to distinguish from hypoplastic MDS morphologically and cytogenetically. Afable *et al.* [59] demonstrated the utility of SNP analysis in AA to complement metaphase cytogenetics for the detection of clonal chromosomal lesions. Combined metaphase cytogenetics and SNP array identified chromosomal lesions in 19% of AA and 54% of hypoplastic MDS. Persistent detection of chromosomal lesions by SNP array would be highly suspicious for hypoplastic MDS and less response to immunotherapy (ATG/cyclosporine) for AA. Therefore, in diagnostically-challenging and equivocal cases of MDS, SNP array can be used to establish the presence of clonal hematopoiesis in patients with normal karyotype and allow appropriate management of the patients.

With the advent of next generation sequencing, SNP arrays are not likely to be used for the identification of genes involved in disease. In the last decade, SNP arrays have played an important role in the identification of individual genes important for the pathogenesis of MDS. *TET2* gene was identified by SNP array genomic profiling and genomic sequencing in 102 patients with MDS, and acquired deletions, missense and nonsense mutations in the *TET2* gene were found in 26% cases of MDS [60]. Recurrent aUPD and microdeletion of chromosome 7q led to identification of *EZH2* gene and mutations in MDS [61,62]. The *TET2* gene mutations have been found to be associated with better response to hypomethylating agents [63], while *EZH2* mutations are poor prognostic marker for MDS. By combining SNP-array and gene expression profiling, Merkerova *et al.* [64] identified *BMP2* and TRIB3 genes located in 20p UPD as potential candidate genes for the pathogenesis of MDS.

## 5. Chronic Myelogenous Leukemia

Chronic myelogenous leukemia (CML) is characterized by the presence of Philadelphia chromosome t(9;22)(q34;q11) (*BCR-ABL1*), for which targeted therapy with tyrosine kinase inhibitors (TKI) have revolutionized the treatment of CML. In an effort to define genetic lesions that cooperate with *BCR-ABL1* to transform to Philadelphia chromosome positive acute leukemia, Mullighan *et al.* [65] studied 304 cases of ALL, including 23 CML cases with a 250K Affymetrix SNP array, and found only 0.47 copy number alterations per case in chronic phase CML (range 0–8), which suggested that chronic phase CML is genomically stable and *BCR–ABL1* is sufficient to induce CML. However, a separate study by Khorashad *et al.* [66] on 10 chronic phase CML patients with high resolution 2.1 million oligonucleotide array comparative genomic hybridization (CGH) showed an average of 53 CNAs per patient (range: 4–166) with majority being amplifications. The difference in CNAs detected by the two techniques is most likely due to the marked difference in resolutions of the arrays. In Mullighan’s study, *IKZF1* was deleted in CML lymphoid blast phase, but not in CML chronic phase [65]. The *IKZF1* encodes *IKAROS* which is an essential transcription factor for normal lymphoid development. Deletion of *IKZF1* results in monoallelic, expression of dominant-negative form, or loss of expression of *IKAROS*. Deletion of *PAX5* and *CDKN2A/B*, together with loss of *IKFZ1* in lymphoid but not myeloid blast phase of CML indicates that these genes play important role in the transformation of CML to lymphoid blast phase of CML [32]. A subset of the CML patients is resistant to TKI therapy, mainly due to *BCR-ABL1* mutations or amplification. In a study to identify genetic alterations in 45 TKI-resistant CML by 250K SNP array, Nowak *et al.* [67] found recurrent submicroscopic alterations, including aUPD in chromosomes 1, 8, 9, 17, 19, and 22. Recurrent deletions of *IGLC1* locus on chromosome 22 were identified in three patients with previous blast crisis, suggesting dedifferentiation into immature progenitors as a possible mechanism of TKI resistance.

## 6. Polycythemia Vera, Essential Thrombocythemia and Primary Myelofibrosis

Polycythemia (Rubra) vera (PV) is a clonal hematopoietic neoplasm characterized by increased red cell mass and *JAK2* V617F mutation in 95% cases, and potential to progress to myelofibrosis or transformation to acute myeloid leukemia. Essential thrombocythemia (ET) is a chronic myeloproliferative neoplasm affecting the megakaryocytic lineage and characterized by abnormally large megakaryocytes in the marrow and persistent thrombocytosis. Primary myelofibrosis (PMF) is characterized by megakaryocytic and myeloid proliferation and progressive fibrosis in the marrow. *JAK2* V617F mutation is identified in approximately 40%–50% cases of ET and approximately 50% of PMF. Of note, *JAK2* V617F mutation can be detected in healthy individuals by high sensitivity methods. CNAs were rare in PV and ET and approximately one third cases of PMF showed small genomic losses (<5 Mb) with 250K SNP array [68]. In terms of copy-neutral aberrations (UPD), recurrent changes were only observed on chromosome 9p. Rice *et al.* [69] confirmed that chromosome 9 abnormalities including 9p LOH, trisomy 9, amplifications of 9p13.3–23.3, 9q33.1–34.13, and 9q34.13 were most frequent in myeloproliferative neoplasms when analyzing 87 myeloproliferative neoplasmas (MPN) cases with an Affymetrix 250K SNP array. Other less frequent recurrent genetic alterations included gains of 1p36.31–36.33, 17q21.2–q21.31, and 17q25.1–25.3, and deletions affecting 18p11.31–11.32. In the study of genetic profiles of myeloproliferative neoplasms by 50K SNP array, Kawamata *et al.* [70] found rare genomic abnormalities in ET, and deletion of chromosomal regions harboring *RB* (13q14) or *NF1* (17q11) in 25% PMF cases. aUPD involving *JAK2* was found in five PV cases with homozygous *JAK2* V617F [70]. A subpopulation with 9p aUPD was detected in 30% of PV and approximately 50% PMF cases, and UPD at 1p was identified in one case of PV. The relationship between *JAK2* V617F mutation and 9p aUPD in PV was further addressed by Wang *et al.* [71]. They investigated 31 PV patients with SNP array and whole genome sequencing and validated the findings in 59 additional PV patients [71]. They defined four PV subgroups based on the quantitative relationship between *JAK2* V617F and 9p aUPD: 42% of patients with heterozygous *JAK2* V617F and no detectable 9p aUPD (subgroup I); 45% of patients with homozygous *JAK2* V617F and an allelic fraction directly proportional to the level of 9p aUPD (subgroup II); 10% with 9p aUPD at approximately twice the level of heterozygous *JAK2* V617F allelic burden (subgroup III) and 3% with trisomy 9p and two copies of *JAK2* V617F allele (subgroup IV), which likely suggest different pathways leading to PV phenotype. These genomic profiling studies indicated that ET and PV are genomically stable and *JAK2* on chromosome 9p is critical for the pathogenesis of MPN. A genome-wide associate study seems to support this, with the identification of a SNP in the *JAK2* locus (rs10974944), which predisposed to the development of *JAK2* V617F-positive myeloproliferative neoplasm [72].

A subset of the patients with MPNs eventually progresses to acute myeloid leukemia. Identification of acquired genetic alterations facilitating this transformation would be valuable for patient stratification. In a study comparing genome profiles of 88 cases of MPN and 71 cases of MPN-blasts phase with 50 and 250 K SNP arrays, leukemic transformation of MPN was accompanied by up to three-fold more genomic alterations per case than chronic phase [73]. The genomic regions commonly affected during leukemic transformation harbored established genes such as *ETV6*, *TP53*, and *RUNX1*, and also new candidate genes on 7q, 16q, 19p, and 21q. Trisomy 8 or amplification of 8q24 (*MYC*) was identified exclusively in *JAK2* V617F(−) MPN-blast phase. A poor prognosis after leukemic transformation was associated with copy number-neutral loss of heterozygosity (CNN-LOH) on either 7q or 9p including homozygous *JAK2* V617F. With higher resolution SNP 6.0 arrays, Rumi *et al.* [74] showed a close relationship between UPD and/or gain of chromosome 9p with progression from PV to post-PV myelofibosis, and genetic aberrations of chromosome 5, 7, or 17p associated with progression to AML and overall survival.

## 7. Myelodysplastic/Myeloproliferative Neoplasms

Myelodysplastic/myeloproliferative neoplasms (MDS/MPN) are characterized by the presence of features of both MDS and MPN. Chronic myelomonocytic leukemia (CMML) is the most common form of MDS/MPN and characterized by persistent monocytosis, dysplasia, and transformation to acute myeloid leukemia. SNP Array 6.0 identified genomic alterations in 60% of patients with CMML with cryptic CN-LOH in 71% and microdeletions in 45% cases [75]. CN-LOH was frequent on 7q harboring *EZH2*, 11q harboring *CBL*, and 4q harboring *TET2*. The presence of multiple chromosomal defects detected by SNP array was associated with a worse overall survival by univariate analysis [75]. In a separate study, UPD occurred in 48% of CMML by 250K SNP array analysis, with the most frequently affected chromosomal region in 11q harboring proto-oncogene *c-CBL* [76]. All patients with UPD 17q and UPD 4q were found to have CMML or M5 primary AML. These studies indicated that CMML is genetically heterogeneous with different pathways to a common disease phenotype, and *CBL* mutations may activate the *RAS* pathway and aberrant *pSTAT5* activation in CMML. A recent study suggested that abnormal SNP array lesions were associated with an inferior complete and partial remission rate, and worse overall survival when compared with patients without SNP lesions after decitabine therapy [77]. This finding, if confirmed, would certainly help better prognostic stratification and treatment of CMML patients.

Juvenile myelomonocytic leukemia (JMML) is a rare clonal hematopoietic disorder of childhood characterized by monocytosis and loss of function of neurofibromatosis 1 (*NF1*) or somatic mutations of genes in *RAS/MAPK* pathway. Flotho *et al.* [78] first applied SNP array on 16 cases of JMML with normal karyotype and identified large regions of UPD on chromosome 17 spanning approximately 55 Mb, which contained the locus of the *NF1* tumor suppressor gene on 17q11.2, in four of five patients with JMML and *NF1*, but not in other cases without *NF1*. Inactivating *NF1* lesion on both alleles was found by mutational analysis in each case. This study indicates that 17q UPD with homozygous loss of normal *NF1* plays a critical role for the pathogenesis of JMML in *NF1* patients.

## 8. Classical Hodgkin Lymphoma

Classical Hodgkin lymphoma (cHL) is characterized by presence of small numbers of neoplastic Reed-Sternberg/Hodgkin cells admixed with mixed inflammatory cells. Metaphase cytogenetic study is typically unsuccessful due to low numbers of neoplastic cells. For the same reason, SNP array is not expected to yield useful information on patient samples. Instead, SNP array 6.0 was performed on cHL cell lines and showed a UPD of chromosome 14q, which was associated with biallelic deletion of *TRAF3* in one cell line, and a gain of copy number for *MAP3K14* in three other cell lines [79]. With primary cHL tissues, interphase cytogenetic analyses confirmed monoallelic deletion of *TRAF3* in 3/20 cases and gains of *MAP3K14* in 5/16 cases. Both *TRAF3* and *MAP3K14* are regulators of the NF-κB pathway, which is constitutively activated in cHL. The study suggested that genetic alterations of the components of the NF-κB pathways contributed to the pathogenesis of cHL, at least in a subset of cases.

A genome-wide association study identified five SNPs on chromosome 6p21.32 associated with nodular sclerosis cHL (NSHL), which is a common subtype of cHL [80]. Two of the SNPs, rs6903608 and rs2858870, were significantly associated with NSHL. The extended haplotype containing these five SNPs was the strongest overall predictor of risk for NSHL [80]. The haplotype with all five risk alleles for the SNPs (Hap3: AGGCT) was associated with a 70% increased risk of NSHL; while the haplotype with all five protective alleles (Hap6: GAATC) was associated with a 60% decreased risk. The DRB1*07:01 allele, which was carried by all individuals with haplotype 6 (GAATC), was associated with a 50% decreased risk of NSHL, suggesting HLA-DRB1 polymorphisms likely implicated in NSHL susceptibility.

## 9. Mature B-Cell Lymphoproliferative Disorder

Mature B-cell neoplasms account for over 90% lymphomas, with the most common types being diffuse large B-cell lymphoma and follicular lymphoma. Diffuse large B-cell lymphoma (DLBCL) is composed of large neoplastic B-cells and genetically heterogeneous. Based on gene expression profiles, DLBCL can be further classified into germinal center B-cell like (GCB) type with good prognosis and activated B-cell like (ABC) type with poor prognosis. Scholtysik *et al.* [81] applied 250K SNP array on 148 cases of DLBCL and found recurrent genomic gains in 24 regions and recurrent genomic losses in 38 regions, with a median of 19 imbalances per case in GCB-DLBCL and 25 per case in ABC-DLBCL. A number of genetic alterations showed different frequencies in GCB and ABC-DLBCL, such as gains of *HDAC7A* on chromosome 12 predominantly in GCB-DLBCL (38% of cases) and losses of *BACH2* and *CASP8AP2* on chromosome 6 predominantly in ABC-DLBCL (35%), suggesting different pathways to lymphomagenesis in the two subtypes of DLBCL. Two new potential tumor suppressor genes *CASP3* and *IL5RA* were identified in the analysis and showed no somatic mutations, suggesting a haploinsufficiency effect of the genes. Another study showed high frequency of LOH over chromosomal region 11p11.2 harboring the gene encoding the protein tyrosine phosphatase receptor type J (*PTPRJ*), which regulates a number of survival pathways [82]. The combination of SNP array and whole exome/whole transcriptome sequencing was especially productive and identified *ARID1B*, *ROBO2*, and *MRS1* as potential tumor suppressor genes and *KLHL6*, *IL31*, and *LRP1* as oncogenes in DLBCL [83]. The impact of genomic alterations on clinical course was studied by 250 SNP array in 124 patients treated with R-CHOP [84]. 20 recurrent genetic lesions were shown to have an impact on the clinical course, in which loss of 8p23.1 had the strongest statistical significance. In this study, five clusters of DLBCL showed distinct genetic profiles, clinical features, and outcomes.

Follicular lymphoma (FL) is a mature B-cell lymphoma composed of malignant germinal center B-cells with some cases eventually transforming to diffuse large B-cell lymphoma. Early study with 10K SNP array on 26 cases of FL revealed recurrent aUDP on 6p, 9p, 12q, and 17p [85]. Homozygosity of 9p and 17p were found predominantly in transformed FL with homozygosity of pre-existing mutation of either *CDKN2A* or *TP53* identified in a subset of cases. Interestingly, 10 cases showed chromosomal regions of homozygosity in FL that were absent in the subsequent transformed FL, suggesting the transformed FL derived from a common malignant precursor but not from step-wise clonal evolution of the preceding FL, at least in some cases [85]. The prognostic significance of aUPD was investigated in 185 cases of FL by 10K SNP array [86]. This study found genetic abnormalities in 65% cases, and more than three abnormalities were associated with inferior overall survival. Recurrent aUPD were detected on 6p, 16p, 12q, 1p36, 10q, and 6q, which were confirmed by other studies [87,88]. O’Shea *et al.* [86] showed that aUPD on 1p36 was correlated with shorter overall survival on multivariate analysis, and aUPD on 16p predicted transformation and poorer progression free survival. Somatic mutations of *TNFRSF14* gene was identified by exon sequencing of the minimum region of deletion of ∼97 kb within 1p36.32, and *TNFRSF14* mutations and 1p36 deletions were associated with inferior overall survival and disease specific survival after adjustment for the International Prognostic Index [89]. Individual genes including *CDKN2A*, *CDKN2B*, *FHIT*, *KIT*, *PEX14*, and *PTPRD*, which were associated with canonical pathways, were implicated by SNP array analysis in FL [87]. FL is characterized by t(14;18)(q32;q21) with *IGH/BCL2* fusion, but a small subset of cases of FL is negative for t(14;18). The genetic profiles of t(14;18) positive and negative FL were compared by SNP array, and showed that gains/amplifications of the *BCL2* gene locus at 18q were only present in the t(14;18)-positive FL [90].

Chronic lymphocytic leukemia is the most common leukemia in adults, and characterized by proliferation of small mature lymphoid cells in the peripheral blood, bone marrow, spleen and lymph nodes. Risk stratification based on FISH findings are routinely performed clinically. Early studies with 10 K and 50 K Affymetrix SNP arrays showed chromosomal imbalances in 65.6% and 82% cases, respectively, including UPD in 20% cases [91,92]. The cytogenetic changes commonly identified by FISH studies including trisomy 12, deletions of *TP53* (17p13), *ATM* (11q22), and *13q14* were readily identified by SNP arrays. SNP arrays found a total of 45 CNAs in 45% cases excluding the four common cytogenetic changes identifiable by FISH [92]. High resolution Affymetrix SNP array 6.0 study on 353 samples of chronic lymphocytic leukemia (CLL) showed similar findings [93], with CN-LOH in 6% of CLL cases, most frequently on 13q, 17p, and 11q. Minimally-deleted regions were identified on 13q14 to the *DLEU1* and *DLEU2* genes, 11q22.3 to *ATM*, 2p16.1-2p15 to a 1.9-Mb fragment containing nine genes, and 8q24.21 to a 486 kb region proximal to the *MYC* locus. Breakpoint cluster regions flanking 13q deletions were found. A 3.5-Mb gain at 2p16 harboring *REL* and *BCL11A* oncogenes and deletion at 6q21 that involved the *AIM1* gene were identified in a subset of cases [91,92], suggesting involvement of these genes in the pathogenesis of CLL. UPD was detected in 7% cases, with 50% involving whole chromosome 13 resulting in homozygous deletion of micro-RNA-15a (*miR-15a*)/*miR-16-1* [92], which was confirmed by another study [94]. The genomic complexity identified by SNP array was an independent risk factor for aggressive CLL and short survival on multivariate analysis [94,95,96,97]. Large genomic aberrations identified by SNP array but not covered by the standard FISH panel was found to be an independent prognosticator of a shorter time to first treatment in CLL, by multivariate analysis [98]. Clonal evolution was shown to developed in 33% patients with unmutated *IGHV*, and in 16% treated patients with mutated *IGHV*, and included known recurrent aberrations such as del(13q) [94]. Analysis of clonal diversity by SNP array for genomic alterations and mosaic distribution of clones was also shown to be predictive of disease progression [99]. Del13q14 is the most frequent cytogenetic abnormality in CLL and associated with good prognosis. Ouillette *et al.* [100] showed that del13q14 was heterogeneous and composed of multiple subtypes, with deletion of *RB* or the *miR15a/miR16* loci as anatomic landmarks. Large (type II) 13q14 deletions spanning the *RB* gene were associated with elevated genomic complexity, accelerated clinical course and short survival [101]. Similarly a separate study was able to classify CLL with del13q14 into two separate clusters characterized by short/biallelic deletion with loss of the *miR-15a/16-1 versus* wide/monoallelic 13q14 deletions [102]. Therefore, despite the established good prognosis of del13q14 by FISH, SNP arrays are clinically useful to identify a subset of cases with del13q14 that has poor prognosis. SNP arrays were shown to be able to supplement FISH studies with unusual signal patterns [103].

Mantle cell lymphoma is an aggressive B-cell lymphoma characterized by *IGH/CCND1* translocation resulting in overexpression of cyclin D1. A large numbers of genomic abnormalities have been identified through metaphase cytogenetics and comparative genomic hybridization in addition to the characteristic t(11;14)(q13;q32). With 250K SNP array, Kawamata *et al.* [104] confirmed the presence of known genetic alterations, including deletion of *INK4A/ARF*, duplication/amplification of *MYC*, deletion of *ATM*, and deletion of *TP53* in 33 samples of MCL. A duplication/amplification at 13q involving oncogenic microRNA, *miR17-92*, other genomic abnormalities, including duplication/amplification of cyclin D1, del(1p), del(6q), dup(3q) and dup(18q), and a number of aUPD sites, including whole chromosome 9 aUPD and 9p aUPD were identified by the SNP array. To identify the target genes in the genomic lesions, Beà *et al.* [105] combined SNP array and gene expression profiling and detected high number of partial UPDs with UPD 17p one of the most common associated with *TP53* gene inactivation. 4 known tumor suppressor genes (*CDKN2C*, *BCL2L11*, *CDKN2A*, and *RB1*) and six new genes (*FAF1*, *MAP2*, *SP100*, *MOBKL2B*, *ZNF280A*, and *PRAME*) were identified in homozygous deletions. The most recurrent amplifications were at 11q13.3–q13.5, 13q31.3, and 18q21.33, targeting *CCND1*, *C13orf25*, and *BCL2*, which may be important for the lymphomogenesis of MCL.

Marginal zone lymphoma is a diverse group of B-cell lymphoma derived from post-germinal center B-cells. SNP array studies in this group of lymphoma are limited. Flossbach *et al.* [106] examined a series of marginal zone B-cell lymphomas of the gastrointestinal tract including ones with large cell transformation by SNP array. They found increase of genomic complexity with lymphoma progression to large cell lymphoma. Gains of protooncogenes such as *REL*, *BCL11A*, *ETS1*, *PTPN1*, *PTEN*, and *KRAS* were identified exclusively in the large cell variants. Copy numbers of *ADAM3A*, *SCAPER* and *SIRPB1* were also associated with progression from small to large cell lymphoma. The gene for tumor necrosis factor alpha-induced protein 3 (*TNFAIP3*, *A20*), a negative regulator of *NF*-κ*B* was found to be deleted at chromosomal region 6q23 in a small subset of marginal zone lymphomas by SNP array analysis [107]. In ocular adnexal MALT lymphoma, one study showed CNAs in approximately 70% cases and UPD detected on 6q (14%) and 3q (10%) [108]. The UPD on 6q likely involves the *A20* gene. In this study, chromosomal gains were most commonly trisomy 3 (31%), trisomy 18 (17%), and 6p and 21q (14%), and the most frequent copy neutral (CN) loss regions were 6q and 9p (7%). Importantly, CNAs were not detected in reactive lymphoid hyperplasia, suggesting that SNP may be useful in difficult cases for diagnostic purpose.

Burkitt lymphoma (BL) is an aggressive B-cell lymphoma mainly in pediatric patients and characterized cytogenetically by t(8;14)(q24;q32) involving the *MYC* gene. Illumina 1M SNP array was applied to 20 cases of BL to identify additional genomic lesions in addition to t(8;14)(q24;q32) [109]. Genomic imbalances were found in 95% cases by SNP array, including recurrent losses of 6q14.1–q22.33, 9p21.3, and 13q14.2–q14.3, gains of 1q23.3–q31.3, chromosome 7, 13q31.3, and partial UPD for 6p12.2-pter, 9p23-pter, and 17p11.2-pter. These genetic alterations resulted in deletion of *CDKN2A* and *TP53* genes, and gains/losses of other genes including *MIR17HG* and *E2F2K* that are involved in the *MYC* pathway, suggesting dysregulation of the *MYC* pathway by 8q24/*MYC* translocation or secondary genomic alterations are essential for development of Burkitt lymphoma.

Hairy cell leukemia (HCL) is an indolent B-cell neoplasm with “hairy cells” in the peripheral blood and bone marrow aspirates, and characteristic *BRAF* V600E mutation. With current therapy, patients with HCL have an excellent prognosis and near normal life span. HCL was found to have a remarkably stable genome by a 250K SNP array analysis [110]. With high resolution SNP Array 6.0, Rinaldi *et al.* [111] confirmed this finding. In this study, the 19 cases of HCL showed an extremely low numbers of CNAs with only two heterozygous losses detected (11%). These studies indicate very limited genetic damages in HCL, which may partly explain the excellent treatment response in current therapy.

Multiple myeloma (MM) is a neoplasm of terminally differentiation B-cells (plasma cells) characterized by multisystem damage and presence of M-Spike in the peripheral blood. In a study of 30 samples of patients with newly diagnosed MM by a 50K SNP array, genomic alterations at 1p, 1q, 6q, 8p, 13, and 16q were most frequent [112]. Multiple regions of UPD were identified for the first time and were found to be interspersed throughout the genome, with a median of three UPD per sample (range, 0–19). These findings were largely confirmed by Agnelli *et al.* [113] in analyzing 41 cases of MM and four cases of plasma cell leukemia. Using unsupervised clustering methods five main groups of genetic imbalances were identified and showed strict correlation with transcriptional expression: cluster I with hyperdiploidy, particularly trisomy 11; cluster II with no or limited alterations; cluster III with 1q gain and chromosome 13 deletion; cluster IV with deletions of 1p, 13, 14, plus deletions of 8p and 22; and cluster V with near-tetraploidy. In an effort to address the prognostic significance of genetic lesions detected by high resolution SNP array, Avet-Loiseau *et al.* [114] showed deletions and amplifications in 98% of patients with MM. Amplifications in 1q and deletions in 1p, 12p, 14q, 16q, and 22q were frequently associated with adverse prognosis, and recurrent amplifications of chromosomes 5, 9, 11, 15, and 19 was associated with a favorable prognosis. Amp(1q23.3), amp(5q31.3), and del(12p13.31) retained independent prognostic value in multivariate analysis. Del(12p13.31) alone, or amp(5q31.3) and del(12p13.31), and high Sβ2M predicted a very poor prognosis. The prognostic significance of 1q amplification was confirmed by two other groups even after removing cases with the most adverse cytogenetic factors such as translocations involving *FGFR3/MMSET*, *MAF*, and *MAFB*, and del17p [115,116]. Walker *et al.* [115] identified UPD on 1q (8%), 16q (9%), and X (20%), that was associated with regions of gain and loss. Kamada *et al.* [116] showed accumulation of deletions and UPD at 22q12.1 associated with poor prognosis in hyperdiploid MM. With a 500K SNP array, Jenner *et al.* [117] identified LOH at 16q involving *CYLD*, a negative regulator of the NF-κB pathway, and *WWOX*, a tumor suppressor involved in apoptosis, that were independently associated with poor prognosis in MM. Multiple myeloma nowadays is typically treated with hypomethylating agent, bortezomib plus melphalan and prednisone. Kim *et al.* [118] showed that increasing genomic complexity identified by SNP arrays correlated with the outcome of the bortezomib plus melphalan and prednisone therapy. Patients with deletion of 1p and gain of 3q did not achieve very good partial response, while complex karyotype and gain of 3q were associated with progressive disease after therapy. Finally, López-Corral *et al.* [119] showed progressive increase in the incidence of CNAs from precursor monoclonal gammopathy of undetermined significance (MGUS) to MM. Gains on 1q, 3p, 6p, 9p, 11q, 19p, 19q, and 21q along with deletions of 1p, 16q, and 22q were significantly less frequent in MGUS than in MM. The frequency of UPD was higher in active MM than in the asymptomatic MM. As expected, the increasing genomic complexity from MGUS to MM is consistent with acquisition of additional genomic lesions as essential pathway to the progression from MGUS to MM.

## 10. Mature T/NK-Cell Lymphoproliferative Disorders

The T/NK-cell lymphoproliferative disorders comprise a diverse group of mature lymphomas or leukemias with variable etiology and clinical course. SNP array studies in this group of neoplasms are limited to a few selected types, largely due to the much lower frequency of occurrence of mature T/NK-cell leukemia/lymphoma overall. Peripheral T-cell lymphoma, not otherwise specified (PTCL, NOS) is the most common T-cell lymphoma and morphologically, phenotypically and cytogenetically heterogeneous. Hartmann et al studied 47 cases of PTCL, NOS with a 250K SNP array, and found genomic alterations in 47% of cases, including recurrent gains of chromosome regions 1q32–43, 2p15–16, 7, 8q24, 11q14–25, 17q11–21 and 21q11–21 and losses of chromosome regions 1p35–36, 5q33, 6p22, 6q16, 6q21–22, 8p21–23, 9p21, 10p11–12, 10q11–22, 10q25–26, 13q14, 15q24, 16q22, 16q24, 17p11, 17p13, and Xp22 [120]. Genomic gains of *REL* gene locus at 2p15–16 and nuclear expression of the *REL* protein by immunohistochemistry were identified in approximately 25% cases of PTCL, NOS, suggesting pathogenetic relevance of *REL* in a subset of PTCL, NOS cases.

Angioimmunoblastic T-cell lymphoma (AILT) is the second most common mature T-cell lymphoma and characterized by proliferation of malignant follicular T-helper cells associated with Epstein-Barr virus infection. In a study comparing the genomic profiles of 40 cases of AILT and 33 cases of PTCL, NOS by 50 K SNP array, three quarters of the cases had relatively stable genomes, while the remaining one quarter had CNAs of various sizes [121]. The presence of CNAs was associated with poor prognosis. Highly-recurrent chromosomal gains in both AILT and PTCL, NOS were clustered at three distinct regions of 8q, 9p, and 19q, and genomic losses at two distinct regions of 3q and 9p. The most common region of LOH was identified in a 440-kb region at 2q32.3. AILT- or PTCL NOS-specific CNAs or LOH were present at 21 regions. Furthermore, overexpression of *CARMA1* at 7p22 and *MYCBP2* at frequently amplified 13q22 predicted poor prognosis. A novel isoform of *IKZF2* was identified in the LOH region at 2q34, which likely acted as a dominant negative form to participate in the transformation to AILT or PTCL, NOS.

Adult T-cell leukemia/lymphoma is a well-defined malignant T-cell neoplasm caused by human T-cell leukemia virus type 1 (HTLV-1). To understand the genetic events occurring after HTLV-1 infection, spectral karyotyping and SNP array of 61 ATLL cases revealed a 2-Mb deletion region breakpoint in 10p11.2 in 35% cases [122]. Transcription Factor 8 (*TCF8*) was identified within this region by gene expression studies as a possible tumor suppressor for ATLL. Loss of *TCF8* resulted in resistance to transforming growth factor β1 (*TGF-β1*) mediated growth inhibition in ATLL cells, which likely contributed to the pathogenesis of ATLL. The same group in a subsequent study showed localization of the breakpoints at 10p11.2 within the *EPC1* locus by SNP array in two cases [123]. *EPC1* is a member of the polycomb group gene family and participate in chromatin formation and gene regulation. *EPC1/ASXL2* and truncated *EPC1* were identified in the two cases respectively, and both were able to induce cellular proliferation in *in vitro* studies, implicating *EPC1* in the pathogenesis of ATLL in some cases.

T-cell prolymphocytic leukemia (T-PLL) is an aggressive T-cell leukemia characterized by proliferation of prolymphocytes in the peripheral blood, bone marrow, liver, spleen and lymph nodes. The most frequent genetic change is inversion of chromosome 14. Gains in 6p (3/12), 8q (10/12), and of losses in 6q (5/12), 8p (7/12), 10p (4/12), 11q (3/12), and 18p (3/12) were identified by a 50K SNP array when analyzing 11 T-PLL with inv(14)(q11q32) or t(14;14)(q11;q32) and one T-PLL case without inv(14)/t(14;14) [124]. Recurrent UPD in 3q, and non-recurrent partial UPD on chromosomes 3, 6, 9, 11, and 13 were identified. In a subsequent study of 18 cases of T-PLL by 250K SNP array, Nowak *et al.* [125] identified abundant copy number alterations, and confirmed the characteristic genetic lesions described before. Recurrent microdeletions targeting *microRNA 34b/c*, *ETS1*, and *FLI1* were implicated in losses in chromosome 11, and *PLEKHA2*, *NBS1*, *NOV* and *MYST3* genes were found to be involved in the breakpoints of chromosome 8. New recurrent lesions were identified on chromosomes 5p, 12p, 13q, 17, and 22 including aUPD on chromosome 17q, with genes *DNAH5*, *ETV6*, *miR-15a*, and *miR-16-1*, *p53*, *BIRC5*, and *SOCS3* implicated in the regions. Future studies of the implicated genes identified by SNP array are likely going to further our understanding of the pathogenesis and provide potential targets for therapy.

Sézary syndrome (SS) is rare but aggressive neoplasm characterized by erythroderma, generalized adenopathy, and infiltrate of cerebriform Sézary cells in the peripheral blood, skin and lymph nodes. A low resolution 10K SNP array of eight patients with SS identified frequent SNP copy number changes and LOH involving 1, 2p, 3, 4q, 5q, 6, 7p, 8, 9, 10, 11, 12q, 13, 14, 16q, 17, and 20 [126]. SNP copy number loss was most frequent at *FAT* gene at 4q35 (75%), followed by *VEGFC* at 4q34.1q34.3 (50%), *NFIB* at chromosome 12 (38%), and *TRIM16* at 17p11.2 (38%). SNP LOH gene clusters at chromosome regions of 9q31q34, 10p11q26, and 13q11q12 were only present in SS but not in normal controls, suggesting their involvement in SS pathology.

## 11. Conclusions

In summary, SNP array studies have contributed significantly in our understanding of the genomics of various hematopoietic malignancies. A summary of the common genomic abnormalities identified by SNP arrays is presented in Table 1. Several oncogenes have been mapped through application of SNP array and were shown to be important in clinical applications. As the cost of next generation sequencing (NGS) continues to drop, NGS will be increasingly applied in clinical labs. As a result, SNP array will gradually phase out. However, as a mature technology with fully developed data analysis and relatively low cost, SNP array will continue to play a role in the clinical lab, especially in situations where diagnostic and prognostic significance of SNP lesions were well established.

**Table 1:** Common genetic abnormalities detected by SNP array in hematopoietic malignancies.

Disease	CNVs/CNAs and/or Associated Genes	LOH/UPD and/or Associated Genes	Prognostic Association	Ref.
B-ALL	Deletion of *PAX5*, *EBF1*, *TCF3*, *LEF1*, *IKZF1* (*IKAROS*), *IKZF3* (*AIOLOS*), *ETV6*, and *CDKN2A/p16INK4A*	9p (*CDKN2A/B*)	-	[26,27,28,29,35]
T-ALL	Deletion of *TAL1*, *RB1*, *PTEN*, *CDKN2A*, *CDKN2B*, *LEF1*, and *STIL*; Gains of *MYB*	9p (*CDKN2A*)	-	[32]
AML	Deletion of 3p14.1–p13 (*FOXP1*, *RYBP*, *FHIT*), 6q27 (*RPS6KA2*), 8q23.3 (*TRPS1*), 10q11.21 (*HNRPF*), 11q25, 12p13.2 (*ETV6*), 15q21.3 (*RFXDC2*), 5q31.1 (*CTNNA1*), 16q22.1 (*CBFB*), 17p13.1 (*TP53*), 17q11.2 (*NF1*)	13q (*FLT3*), 11p (WT1, PU1) and 11q (MLL), 19q (*CEBPA*), 6p and 21q (*RUNX1*)	Worse prognosis: ≥2 genomic lesions detected by SNP array	[42,43,44,45,47,48,49]
	Amplifications of 8q23.2 (*MYC*), 11q23.3 (*MLL*), and 21q22.2 (*ETS2*)		*P53* mutations, or *P53* mutations coupled with 17p-LOH; Genomic lesions including aUPD identified by SNP array	
MDS	Deletion and aUPD of chromosomes 1, 5q, 7, 11, 17, and 21		Worse prognosis: UPDs of 7q; New genetic lesions detected by SNP array; *EZH2* mutations	[57,58,60,61,62,63,64]
	Deletion of *EZH2* and *TET2*			
	UPD 20p (*BMP2* and *TRIB3*)		Favorable prognosis: *TET2* mutations (better response to hypomethylating agents)	
CML	Frequent amplifications in chronic phase	1, 8, 9, 17, 19, and 22 in TKI-resistant CML	-	[66,67]
	Deletion of IKFZ1 in lymphoid blast phase			
MPN	Rare in ET and PV	9p (JAK2)	Worse prognosis: CNN-LOH on 7q or 9p (*JAK2* V617F); Genetic aberrations of chromosome 5, 7, or 17p	[68,69,70,73,74]
	Deletion of 13q14 (*RB*) or 17q11 (*NF1*) in PMF			
CMML	Frequent microdeletions	7q (*EZH2*), 11q (*CBL*), and 4q (*TET2*)	Worse prognosis: Multiple chromosomal defects detected by SNP array	[75,76]
cHL	Gain of *MAP3K14*	14q (*TRAF3*)	-	[79]
DLBCL	Frequent gains and deletions; gains *HDAC7A* on chromosome 12 predominantly in GCB-DLBCL, losses of *BACH2* and *CASP8AP2* on chromosome 6 predominantly in ABC-DLBCL; Potential tumor suppressor genes: *CASP3*, *IL5RA ARID1B*, *ROBO2* and *MRS1*; Potential oncogenes: *KLHL6*, *IL31* and *LRP1*	11p11.2 (*PTPRJ*),	Worse prognosis: Loss of 8p23.1	[81,82,83,84]
FL	*CDKN2A*, *CDKN2B*, *FHIT*, *KIT*, *PEX14*, and *PTPRD*	1p36 (*TNFRSF14*), 6p, 6q, 9p (*CDKN2A*), 10q, 12q, 16p, and 17p (*TP53*)	Worse prognosis: >3 SNP abnormalities; aUPD and deletion of 1p36, aUPD of 16p	[85,86]
CLL	Deletion of 17p13 (*TP53*), 11q22 (*ATM*) and 13q14 (*DLEU1* and *DLEU2*), 2p16.1–2p15, 8q24.21, 6q21 (*AIM1*)	13q, 13 (*miR-15a/miR-16-1*), 17p, and 11q	Worse prognosis: Genomic complexity; large genomic aberrations; large (type II) 13q14 deletions	[91,92,93,101]
	Gain of 12, 2p16 (*REL*, *BCL11A*)			
MCL	Deletion of *INK4A/ARF*, *ATM*, *TP53*, 1p, 6q, *CDKN2C*, *BCL2L11*, *CDKN2A*, and *RB1*, *FAF1*, *MAP2*, *SP100*, *MOBKL2B*, *ZNF280A*, and *PRAME.*	9p, 9, 17p (*TP53*)	-	[104,105]
	Amplification of *MYC*, 11q13(*cyclin D1*), 13q (*miR17-92*, *C13 or f25*), dup(3q), 18q (*BCL2*)			
MZL	Deletion of 6q23 (*TNFAIP3*, *A20*), 9p	6q (*A20*), 3q	-	[106,107,108]
	Gain of 3, 18, 6p and 21q			
	Gains of REL, BCL11A, ETS1, PTPN1, PTEN and KRAS in transformation to DLBCL			
BL	Losses of 6q14.1–q22.33, 9p21.3 (*CDKN2A*), and 13q14.2–q14.3	6p12.2-pter, 9p23-pter, and 17p11.2-pter (*TP53*).	-	[109]
	Gains of 1q23.3–q31.3, 7, 13q31.3,			
MM	Genomic alterations at 1p, 1q, 6q, 8p, 13, and 16q	1q, 16q (*CYLD*), and X	Worse prognosis: Amplifications in 1q and deletions in 1p, 12p, 14q, 16q, and 22q	[111,112,115,116,117]
			UPD of 16q (*CYLD*)	
			Favorable prognosis: Amplifications of 5, 9, 11, 15, and 19	
PTCL, NOS	Losses of 1p35-36, 3q, 5q33, 6p22, 6q16, 6q21–22, 8p21–23, 9p21, 10p11–12, 10q11-22, 10q25–26, 13q14, 15q24, 16q22, 16q24, 17p11, 17p13 and Xp22	2q32.3	-	[120,121]
	Gains of 1q32–43, 2p15–16 (*REL*), 7, 8q24, 11q14–25, 17q11–21 and 21q11–21, 9p and 19q			
AILT	Loss of 3q and 9p	2q32.3	Worse prognosis: The presence of CNAs; overexpression of CARMA1 at 7p22 and MYCBP2 at 13q22	[121]
	Gains of 8q, 9p and 19q			
ATLL	Deletion of 10p11.2 ( *TCF8*)	-	-	[122]
T-PLL	Loss in 6q, 8p, 10p, 11q (*microRNA 34b/c*, *ETS1* and *FLI1)*, and 18p and Gains of 6p, 8q	3q, 17q	-	[124,125]
	Aberrations in 5p, 12p, 13q, 17 and 22 (*DNAH5*, *ETV6*, *miR-15a* and *miR-16-1*, *p53*, *BIRC5*, and *SOCS3*)			
SS	Loss at 4q35 (*FAT*), 4q34 (*VEGFC*), 12 (*NFIB*), and 17p11.2 (*TRIM16*)	9q31q34, 10p11q26, and 13q11q12	-	[126]

CNV, copy number variations; CAN, copy number aberrations; LOH, loss of heterozygosity; UPD, uniparental disomy; Ref, reference; B-ALL, B lymphoblastic leukemia; T-ALL, T lymphoblastic leukemia; AML, acute myeloid leukemia; MDS, myelodysplastic syndrome; CML, chronic myelogenous leukemia; MPN, myeloproliferative neoplasm; PV, polycythemia (Rubra) vera; ET, essential thrombocythemia; PMF, primary myelofibrosis; CMML, chronic myelomonocytic leukemia; cHL, classical Hodgkin lymphoma; DLBCL, diffuse large B-cell lymphoma; FL, follicular lymphoma; CLL, chronic lymphocytic leukemia; MCL, mantle cell lymphoma; MZL, marginal zone lymphoma; BL, Burkitt lymphoma; MM, multiple myeloma; PTCL, NOS, peripheral T-cell lymphoma, NOS; AILT, angioimmunoblastic T-cell lymphoma; ATLL, adult T-cell leukemia/lymphoma; T-PLL, T-cell prolymphocytic leukemia; SS, Sezary syndrome.
